# Crystal structures of two 2,9-di­thia-13-aza­dispiro­[4.1.4^7^.3^5^]tetra­decan-6-ones

**DOI:** 10.1107/S2056989015020885

**Published:** 2015-11-21

**Authors:** Vijayan Viswanathan, Shanmugavel Bharkavi, Subbu Perumal, Devadasan Velmurugan

**Affiliations:** a Centre of Advanced Study in Crystallography and Biophysics, University of Madras, Guindy Campus, Chennai 600 025, India; b Department of Organic Chemistry, School of Chemistry, Madurai Kamaraj University, Madurai 625 021, India

**Keywords:** crystal structure, 2,9-di­thia-13-aza­dispiro­[4.1.4^7^.3^5^]]tetra­decan-6-one, thio­phene, piperidine, di­spiro: hydrogen bonding

## Abstract

In the title compounds 4,11-dihy­droxy-13-methyl-1,8-di-*p*-tolyl-2,9-di­thia-13- aza­dispiro­[4.1.4^7^.3^5^]tetra­decan-6-one, C_26_H_31_NO_3_S_2_, (I), and 13-benzyl-4,11-dihy­droxy-1,8-bis­(4-methyl­phen­yl)-2,9-di­thia-13-aza­dispiro­[4.1.4^7^.3^5^]tetradecan-6-one, C_32_H_35_NO_3_S_2_, (II), the piperidine rings adopt distorted chair conformations. The thio­phene rings in (I) have envelope conformations, with the spiro C atoms as the flaps. In (II), one thio­phene ring (*D*) has an envelope conformation, with the hy­droxy-substituted C atom as the flap, while the other thio­phene ring (*E*) has a twisted conformation on the C—C bond involving the spiro C atom and the toluyl-substituted C atom. In (I), the mean plane of the piperidine ring makes dihedral angles of 75.16 (9) and 73.33 (8)° with the mean planes of the thio­phene rings (*D* and *E*), respectively. In (II), the corresponding dihedral angles are 70.95 (11) and 77.43 (12)°. In both compounds, there is an intra­molecular O—H⋯O hydrogen bond forming an *S*(6) ring motif. In the crystal of (I), mol­ecules are linked *via* O—H⋯N and C—H⋯O hydrogen bonds, forming chains along [010]. There are also π–π inter­actions present involving inversion-related benzene rings, linking the chains to form slabs parallel to (100). In the crystal of (II), mol­ecules are linked *via* O—H⋯O hydrogen bonds, forming inversion dimers with an *R*_4_^4^(8) ring motif. The dimers are linked by C—H⋯π inter­actions, forming slabs parallel to (001).

## Abstract 2

The title compounds, (I) and (II), differ only in the substituent on the N atom of the central piperidine ring; methyl in (I) and benzyl in (II). In each mol­ecule, the 4,11-dihy­droxy groups are involved in intra­molecular O—H⋯O hydrogen bonds. In the crystal of (I), mol­ecules are linked *via* O—H⋯N hydrogen bonds, forming chains along the *b*-axis direction. In the crystal of (II), mol­ecules are linked *via* O—H⋯O hydrogen bonds, forming inversion dimers with an 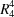
(8) ring motif.

## Chemical context   

Piperidine derivatives have had an important impact in the medical field due to their wide variety of pharmacological activities, and they form an essential part of the mol­ecular structure of important drugs (Hema *et al.*, 2005*a* ▸,*b* ▸). Piperidine derivatives are used clinically to prevent post-operative vomiting, to speed up gastric emptying before anaesthesia or to facilitate radiological evaluation, and to correct a variety of disturbances of gastro-intestinal functions (Hema *et al.*, 2005*a* ▸,*b* ▸). The piperidine structural motif is present in natural alkaloids (Raghuvarman *et al.*, 2014 ▸). Notably it is found in the fire ant toxin solenopsin and is an inhibitor of phosphatidyl­inositol-3- kinase signalling and angiogenesis (Rajalakshmi *et al.*, 2012 ▸). Piperidines are known to have CNS depressant action at low dosage levels and stimulant activity with increased doses. They have been used as anti­tumor (Nguyen Thi Thanh *et al.*, 2014 ▸), anti­microbial (Perumal *et al.*, 2014 ▸), anti­fungal, hypoglycaemic, hypolipidemic, anti-acetyl cholin­esterase (Singh *et al.*, 2009 ▸), anti-coagulant (Mochizuki *et al.*, 2008 ▸), anti­histamines, anaesthetics, tranquilizers, analgesic, ganglionic blocking and as hypotensive agents (Pandey & Chawla, 2012 ▸). The properties of piperidine derivatives depends on the nature of the side groups and their orientations. As part of our studies in this area, we have synthesized two new 2,9-di­thia-13-aza­dispiro­[4.1.4^7^.3^5^]tetra­decan-6-one derivatives, each incorporating a piperidine ring, and report herein on their crystal structures.
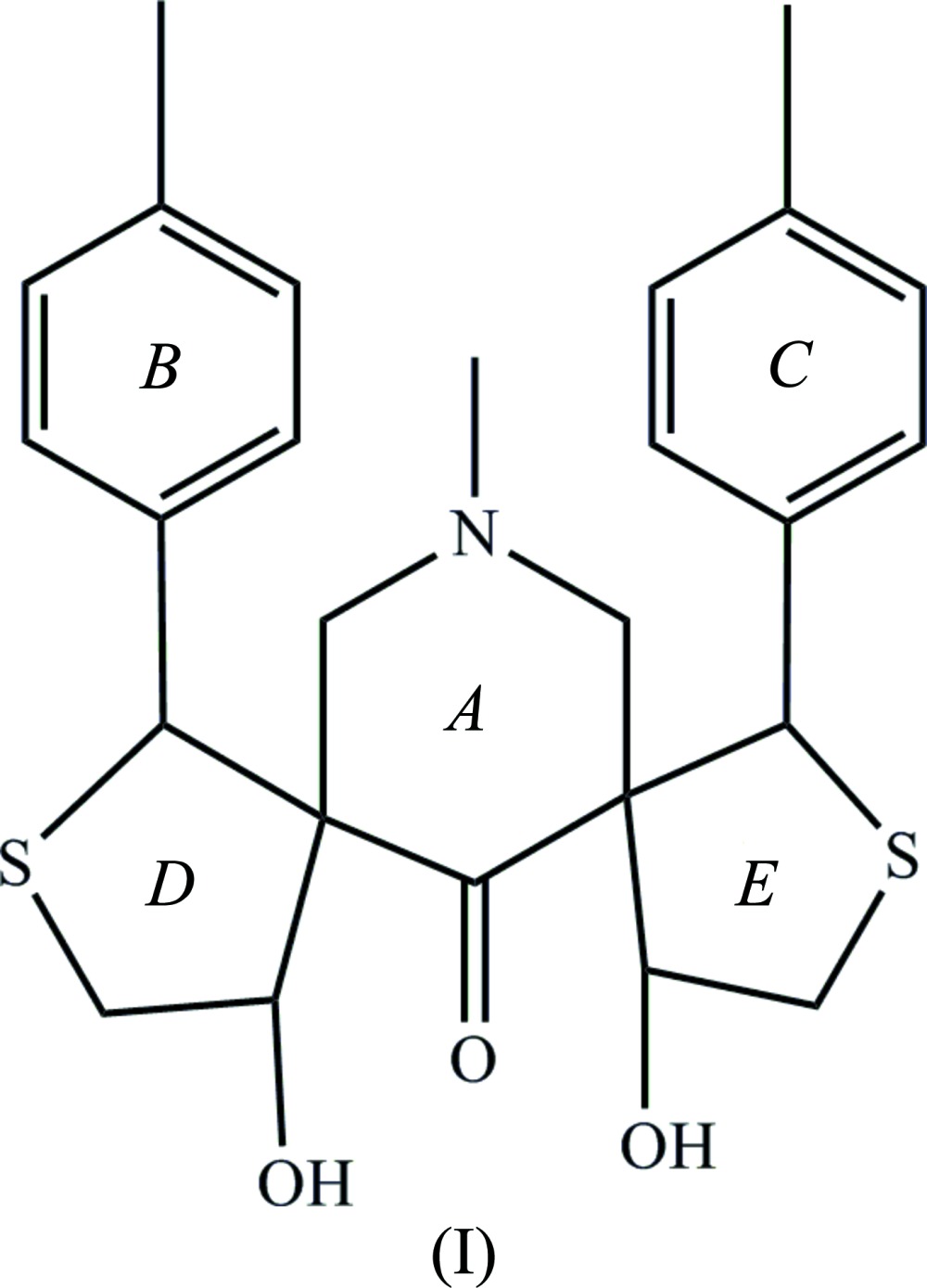
 
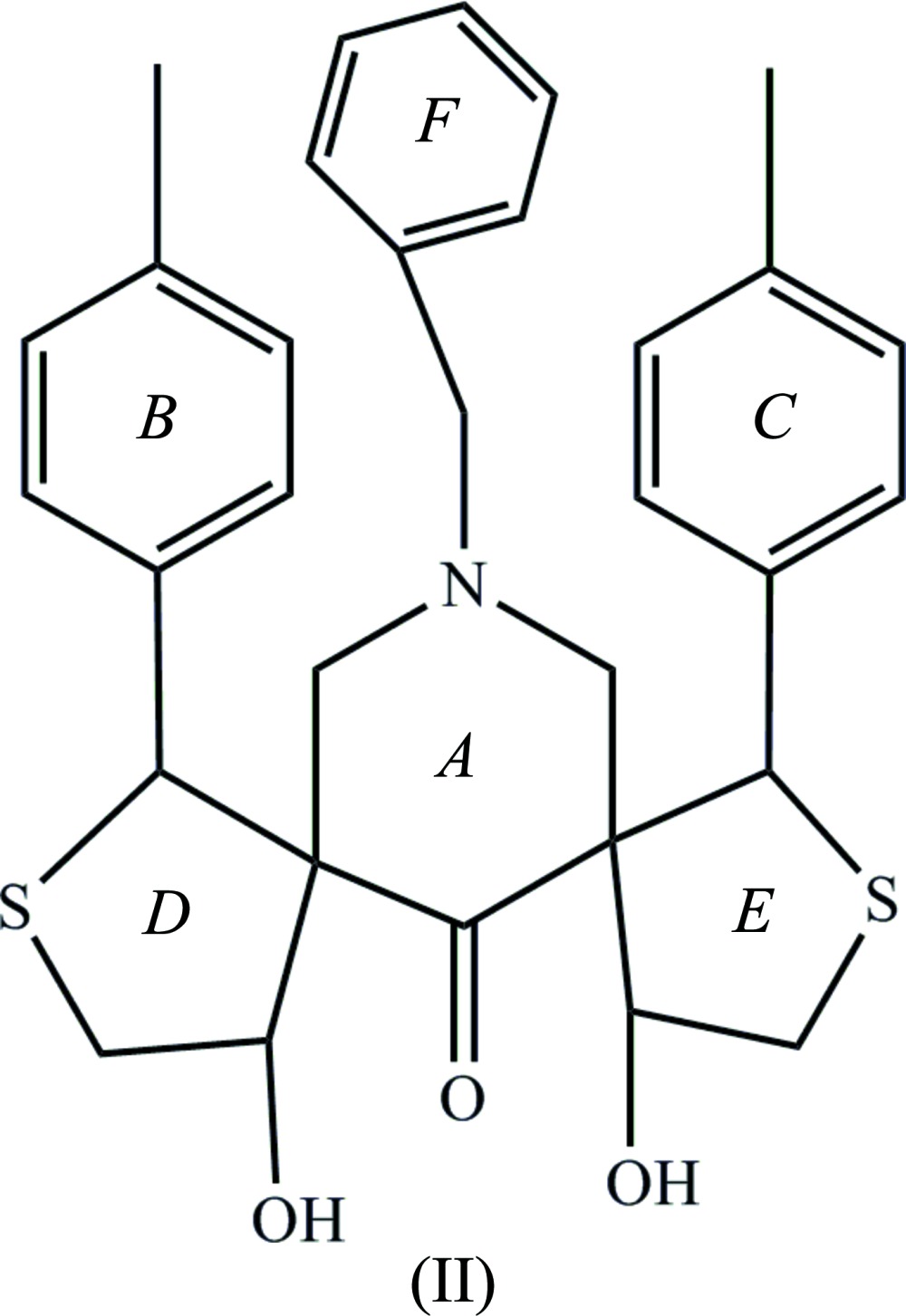


## Structural commentary   

**Figure 1:**
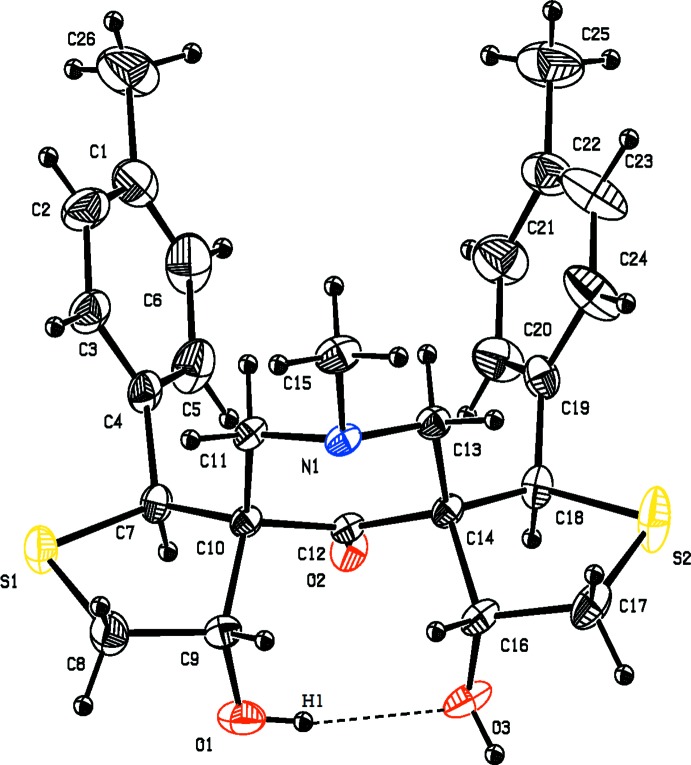
The mol­ecular structure of compound (I), showing the atom labelling. Displacement ellipsoids are drawn at the 30% probability level.

**Figure 3:**
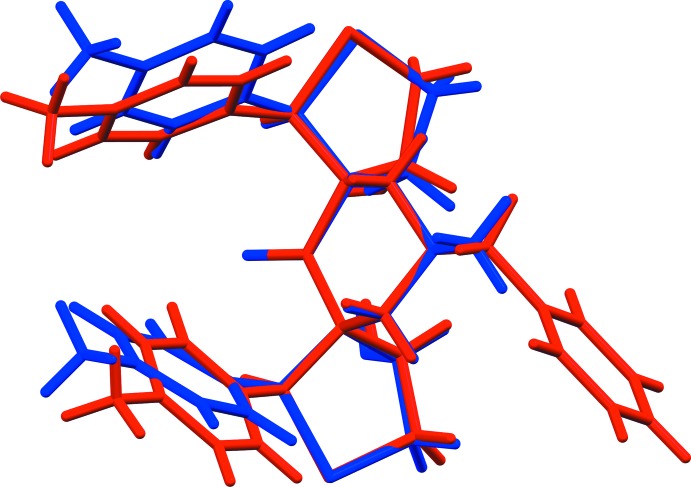
A view of the structural overlay of compounds (I) and (II) [compound (I) is blue and compound (II) is red].

The mol­ecular structure of compounds, (I) and (II), are shown in Figs. 1 ▸ and 2 ▸, respectively. A view of the structural overlay of the two compounds is shown in Fig. 3 ▸. The essential differences appear to be related to the orientations of the toluyl substituents, *viz*. rings *B* an *C*.

In both mol­ecules there is an intra­molecular O—H⋯O hydrogen bond present forming an *S*(6) ring motif. Most piperidine derivatives are known to have chair conformations (Sekar & Parthasarathy *et al.*, 1993 ▸). The title compounds are no exception and the piperidine rings (*A* = C10–C14/N1) adopt distorted chair conformations in both compounds. In compound (I), atoms C12 and N1 are displaced from the mean plane through the four other almost planar atoms (C10/C11/C13/C14) by −0.4543 (15) and 0.7047 (13) Å, respectively. In (II) it is atoms C14 and N1 that are displaced from the mean plane through the four other planar atoms (C10*-*-C13), by 0.412 (2) and −0.7543 (18) Å, respectively.

In compound (I), the thio­phene rings *D* (C7–C10/S1) and *E* (C14/C16–C18/S2) have envelope conformations with atoms C10 and C14, respectively, as the flaps. They deviate from the mean plane through the four other atoms in the ring by 0.6277 (15) Å for C10 and 0.6494 (15) Å for C14. The mean plane of the piperidine ring *A* makes dihedral angles of 75.16 (9) and 73.33 (8)° with the mean planes of the thio­phene rings *D* and *E*, respectively. The mean plane of thio­phene ring *D* makes a dihedral angle of 60.10 (1)° with toluyl ring *B* (C1–C6), and the mean plane of thio­phene ring *D* make a dihedral angle of 58.14 (1)° with toluyl ring *C* (C19–C24). Rings *B* and *C* are inclined to one another by 66.39 (13)°.

In compound (II), thio­phene ring *D* (C7–C10/S1) has an envelope conformation with atom C9 as the flap. It deviates from the mean plane through the other four atoms by 0.621 (2) Å. Thio­phene ring *E* (C13/C15–C17/S2) has a twisted conformation on the C13—C17 bond. These two atoms deviate from the plane (C15/C16/S2) by 0.291 (2) and −0.490 (2) Å, respectively. The piperidine ring *A* mean plane makes dihedral angles of 70.95 (11) and 77.43 (12)° with the mean planes of thio­phene rings *D* and *E*, respectively. The mean plane of thio­phene ring *D* make a dihedral angle of 52.42 (1)° with toluyl ring *B* (C1–C6), and the mean plane of thio­phene ring *D* make a dihedral angle of 65.71 (1)° with toluyl ring *C* (C18–C23). Benzyl ring *F* (C25–C30) makes a dihedral angle of 75.09 (1)° with the mean plane of piperidine ring *A*. Rings *B* and *C* are inclined to one another by 74.33 (12)°.

## Supra­molecular features   

**Figure 4:**
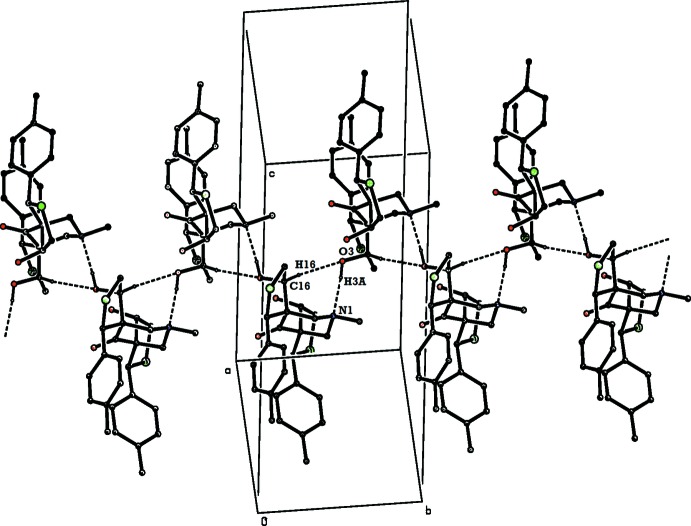
The crystal packing of the compound (I), illustrating the formation of chains along [010]. Hydrogen bonds are shown as dashed lines (see Table 1 ▸). H atoms not involved in hydrogen bonding have been omitted for clarity.

**Figure 5:**
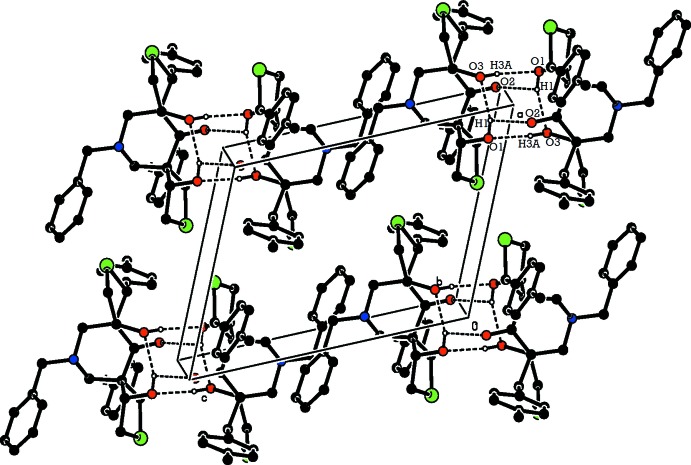
A partial view of the crystal packing of compound (I), showing the π–π inter­action (dashed line), involving inversion-related toluyl rings. H atoms not involved in this inter­action have been omitted for clarity and the centroids are shown as small red balls.

In the crystal of (I), mol­ecules are linked *via* O—H⋯N and C—H⋯O hydrogen bonds, forming chains along the *b-*axis direction (Table 1 ▸ and Fig. 4 ▸). The chains are linked *via* weak π–π stacking inter­actions involving inversion-related *C* toluyl rings [centroid-to-centroid distance of 3.9582 (17) Å; Fig. 5 ▸], forming slabs parallel to the *bc* plane.

**Figure 6:**
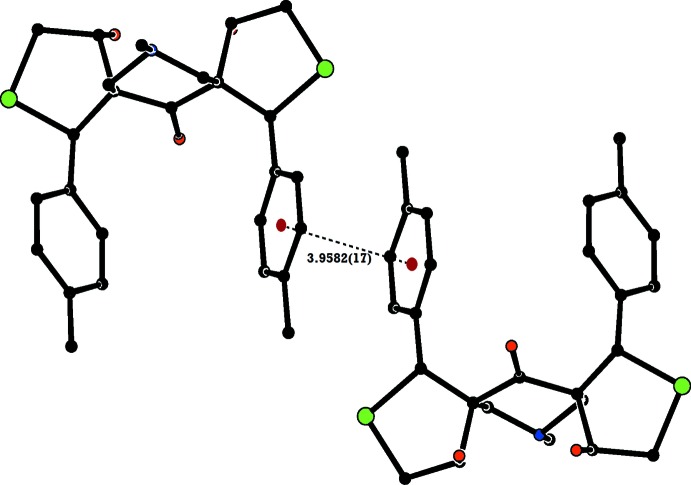
The crystal packing of compound (II), viewed along the *b* axis. Hydrogen bonds are shown as dashed lines (see Table 2 ▸ for details). H atoms not involved in hydrogen bonding have been omitted for clarity.

**Figure 7:**
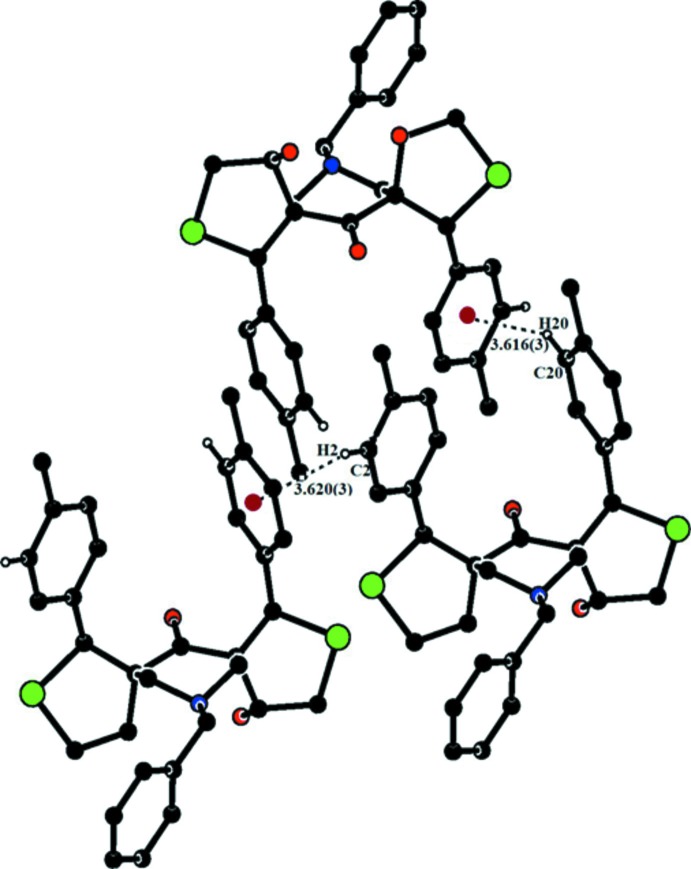
A partial view of the crystal packing of compound (II), showing the C—H⋯π inter­actions as dashed lines (see Table 2 ▸ for details). H atoms not involved in these inter­actions have been omitted for clarity and the centroids are shown as small red balls.

In the crystal of (II), mol­ecules are linked *via* O—H⋯O hydrogen bonds, forming inversion dimers enclosing an 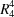
(8) ring motif (Table 2 ▸ and Fig. 6 ▸). There are C—H⋯π inter­actions present (Fig. 7 ▸) linking the dimers to form slabs parallel to the *ab* plane.

## Database survey   

A search of the Cambridge Structural Database (Version 5.36, last update May 2015; Groom & Allen, 2014 ▸) for the sub-structure 2,9-di­thia-13-aza­dispiro­[4.1.4^7^.3^5^]tetra­decan-6-one gave zero hits.

## Synthesis and crystallization   

**Compound (I)**: A mixture of (3*E*,5*E*)-1-methyl-3,5-bis­(4-methyl­benzyl­idene)piperidin-4-one (1 mmol) 1, 1,4-di­thiane-2,5-diol (1 mmol) 2 and tri­ethyl­amine (0.25 eq) in di­chloro­methane (6 ml) was heated under reflux for 3 h. After completion of the reaction (TLC), the solvent was removed and the product was purified by flash column chromatography using a petroleum ether–ethyl acetate mixture (4:1 v/v) as eluent to afford pure state of the title compound. After purification the compound was recrystallized in CHCl_3_ by slow evaporation.

**Compound (II)**: A mixture of (3*E*,5*E*)-1-benzyl-3,5-bis­(4-methyl­benzyl­idene)piperidin-4-one (1 mmol) 1, 1,4-di­thiane-2,5-diol (1 mmol) 2 and tri­ethyl­amine (0.25 eq) in di­chloro­methane (6 ml) was heated under reflux for 3 h. After completion of the reaction (TLC), the solvent was removed and the product was purified by flash column chromatography using a petroleum ether–ethyl acetate mixture (4:1 v/v) as eluent to afford pure state of the title compound. After purification the compound was recrystallized in CHCl_3_ by slow evaporation.

## Refinement   

Crystal data, data collection and structure refinement details are summarized in Table 3 ▸. The hy­droxy H atoms were located in difference Fourier maps. For compound (II), the hy­droxy H atom, H3*A*, was freely refined. Those of compound (I) and the second hy­droxy H atom in compound (II) were refined as riding: O—H = 0.82 Å with *U*_iso_(H) = 1.5*U*_eq_(O). The C-bound hydrogen atoms were placed in calculated positions and refined as riding: C—H = 0.93–0.98 Å with *U*_iso_(H) = 1.5*U*_eq_(C) for methyl H atoms and 1.2*U*_eq_(C) for other H atoms.

## Supplementary Material

Crystal structure: contains datablock(s) global, I, II. DOI: 10.1107/S2056989015020885/su5199sup1.cif

Structure factors: contains datablock(s) I. DOI: 10.1107/S2056989015020885/su5199Isup2.hkl

Structure factors: contains datablock(s) II. DOI: 10.1107/S2056989015020885/su5199IIsup3.hkl

Supporting information file. DOI: 10.1107/S2056989015020885/su5199Isup4.cml

Supporting information file. DOI: 10.1107/S2056989015020885/su5199IIsup5.cml

CCDC references: 1435024, 1435023

Additional supporting information:  crystallographic information; 3D view; checkCIF report

## Figures and Tables

**Figure 2:**
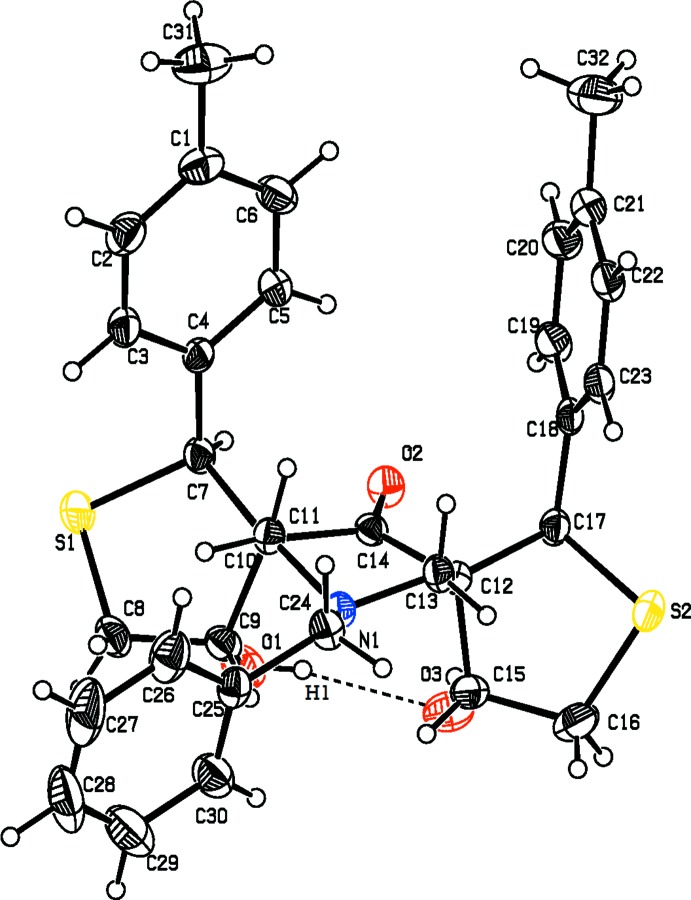
The mol­ecular structure of compound (II), showing the atom labelling. Displacement ellipsoids are drawn at the 30% probability level.

**Table 1:** Hydrogen-bond geometry (Å, °) for (I)

*D*—H⋯*A*	*D*—H	H⋯*A*	*D*⋯*A*	*D*—H⋯*A*
O1—H1⋯O3	0.82	2.16	2.955 (2)	163
O3—H3*A*⋯N1^i^	0.82	1.99	2.798 (2)	167
C16—H16⋯O3^ii^	0.98	2.39	3.273 (2)	150

Symmetry codes: (i) 
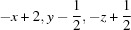
; (ii) 
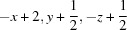
.

**Table 2:** Hydrogen-bond geometry (Å, °) for (II) *Cg*4 and *Cg*5 are the centroids of the *B* (C1–C6) and *C* (C18–C23) toluyl rings, respectively.

*D*—H⋯*A*	*D*—H	H⋯*A*	*D*⋯*A*	*D*—H⋯*A*
O1—H1⋯O3	0.82	2.09	2.873 (3)	159
O3—H3*A*⋯O1^i^	0.82 (6)	2.06 (5)	2.880 (3)	174 (1)
C2—H2⋯*Cg*5^ii^	0.93	2.80	3.620 (3)	148
C20—H20⋯*Cg*4^iii^	0.93	2.79	3.616 (3)	149

Symmetry codes: (i) 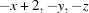; (ii) 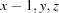; (iii) 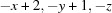.

**Table 3:** Experimental details

	(I)	(II)
Crystal data		
Chemical formula	C_26_H_31_NO_3_S_2_	C_32_H_35_NO_3_S_2_
*M*_r_	469.64	545.73
Crystal system, space group	Monoclinic, *P*2_1_/*c*	Triclinic, *P* 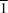
Temperature (K)	293	293
*a*, *b*, *c* (Å)	10.7160 (8), 8.5570 (5), 25.6960 (3)	9.9803 (6), 11.7773 (8), 13.6506 (14)
α, β, γ (°)	90, 92.374 (5), 90	105.524 (5), 107.215 (5), 103.087 (4)
*V* (Å^3^)	2354.2 (2)	1392.90 (19)
*Z*	4	2
Radiation type	Mo *K*α	Mo *K*α
μ (mm^−1^)	0.26	0.23
Crystal size (mm)	0.23 × 0.16 × 0.10	0.20 × 0.15 × 0.10

Data collection		
Diffractometer	Bruker SMART APEXII area detector	Bruker SMART APEXII area detector
Absorption correction	Multi-scan (*SADABS*; Bruker, 2008 ▸)	Multi-scan (*SADABS*; Bruker, 2008 ▸)
*T*_min_, *T*_max_	0.944, 0.975	0.956, 0.978
No. of measured, independent and observed [*I* > 2σ(*I*)] reflections	21401, 5871, 4793	20239, 5668, 4271
*R*_int_	0.023	0.035
(sin θ/λ)_max_ (Å^−1^)	0.668	0.626

Refinement		
*R*[*F* ^2^ > 2σ(*F* ^2^)], *wR*(*F* ^2^), *S*	0.052, 0.141, 1.02	0.045, 0.136, 1.07
No. of reflections	5871	5668
No. of parameters	294	349
No. of restraints	0	1
H-atom treatment	H-atom parameters constrained	H atoms treated by a mixture of independent and constrained refinement
Δρ_max_, Δρ_min_ (e Å^−3^)	0.51, −0.73	0.30, −0.50

Computer programs: *APEX2* and *SAINT* (Bruker, 2008 ▸), *SHELXS97* and *SHELXL97* (Sheldrick, 2008 ▸), *ORTEP-3 for Windows* (Farrugia, 2012 ▸), *Mercury* (Macrae *et al.*, 2008 ▸) and *PLATON* (Spek, 2009 ▸).

## supplementary crystallographic information

### (I) 4,11-Dihydroxy-13-methyl-1,8-di-p-tolyl-2,9-dithia-13-azadispiro[4.1.47.35]tetradecan-6-one Crystal data

**Table:**

C_26_H_31_NO_3_S_2_	*F*(000) = 1000
*M**_r_* = 469.64	*D*_x_ = 1.325 Mg m^−^^3^
Monoclinic, *P*2_1_/*c*	Mo *K*α radiation, λ = 0.71073 Å
Hall symbol: -P 2ybc	Cell parameters from 5871 reflections
*a* = 10.7160 (8) Å	θ = 1.6–28.4°
*b* = 8.5570 (5) Å	µ = 0.26 mm^−^^1^
*c* = 25.6960 (3) Å	*T* = 293 K
β = 92.374 (5)°	Block, colourless
*V* = 2354.2 (2) Å^3^	0.23 × 0.16 × 0.10 mm
*Z* = 4	

### (I) 4,11-Dihydroxy-13-methyl-1,8-di-p-tolyl-2,9-dithia-13-azadispiro[4.1.47.35]tetradecan-6-one Data collection

**Table:**

Bruker SMART APEXII area-detector diffractometer	5871 independent reflections
Radiation source: fine-focus sealed tube	4793 reflections with *I* > 2σ(*I*)
Graphite monochromator	*R*_int_ = 0.023
ω and φ scans	θ_max_ = 28.4°, θ_min_ = 1.6°
Absorption correction: multi-scan (*SADABS*; Bruker, 2008)	*h* = −14→11
*T*_min_ = 0.944, *T*_max_ = 0.975	*k* = −11→10
21401 measured reflections	*l* = −34→33

### (I) 4,11-Dihydroxy-13-methyl-1,8-di-p-tolyl-2,9-dithia-13-azadispiro[4.1.47.35]tetradecan-6-one Refinement

**Table:**

Refinement on *F*^2^	Primary atom site location: structure-invariant direct methods
Least-squares matrix: full	Secondary atom site location: difference Fourier map
*R*[*F*^2^ > 2σ(*F*^2^)] = 0.052	Hydrogen site location: inferred from neighbouring sites
*wR*(*F*^2^) = 0.141	H-atom parameters constrained
*S* = 1.02	*w* = 1/[σ^2^(*F*_o_^2^) + (0.0662*P*)^2^ + 1.3213*P*] where *P* = (*F*_o_^2^ + 2*F*_c_^2^)/3
5871 reflections	(Δ/σ)_max_ = 0.003
294 parameters	Δρ_max_ = 0.51 e Å^−^^3^
0 restraints	Δρ_min_ = −0.73 e Å^−^^3^

### (I) 4,11-Dihydroxy-13-methyl-1,8-di-p-tolyl-2,9-dithia-13-azadispiro[4.1.47.35]tetradecan-6-one Special details

**Table:**

Geometry. All esds (except the esd in the dihedral angle between two l.s. planes) are estimated using the full covariance matrix. The cell esds are taken into account individually in the estimation of esds in distances, angles and torsion angles; correlations between esds in cell parameters are only used when they are defined by crystal symmetry. An approximate (isotropic) treatment of cell esds is used for estimating esds involving l.s. planes.
Refinement. Refinement of F^2^ against ALL reflections. The weighted R-factor wR and goodness of fit S are based on F^2^, conventional R-factors R are based on F, with F set to zero for negative F^2^. The threshold expression of F^2^ > 2sigma(F^2^) is used only for calculating R-factors(gt) etc. and is not relevant to the choice of reflections for refinement. R-factors based on F^2^ are statistically about twice as large as those based on F, and R- factors based on ALL data will be even larger.

### (I) 4,11-Dihydroxy-13-methyl-1,8-di-p-tolyl-2,9-dithia-13-azadispiro[4.1.47.35]tetradecan-6-one Fractional atomic coordinates and isotropic or equivalent isotropic displacement parameters (Å^2^)

**Table:**

	*x*	*y*	*z*	*U*_iso_*/*U*_eq_	
C1	0.4048 (2)	0.3240 (4)	0.03800 (10)	0.0719 (8)	
C2	0.3926 (2)	0.4410 (4)	0.07393 (10)	0.0664 (7)	
H2	0.3493	0.5313	0.0642	0.080*	
C3	0.44302 (18)	0.4284 (3)	0.12434 (9)	0.0505 (5)	
H3	0.4324	0.5097	0.1478	0.061*	
C4	0.50904 (16)	0.2962 (2)	0.14024 (7)	0.0398 (4)	
C5	0.5191 (2)	0.1764 (3)	0.10455 (10)	0.0628 (7)	
H5	0.5603	0.0847	0.1144	0.075*	
C6	0.4687 (3)	0.1914 (4)	0.05435 (11)	0.0801 (9)	
H6	0.4781	0.1099	0.0309	0.096*	
C7	0.57099 (15)	0.2778 (2)	0.19388 (7)	0.0333 (3)	
H7	0.5748	0.1657	0.2015	0.040*	
C8	0.61777 (19)	0.4164 (3)	0.28561 (8)	0.0506 (5)	
H8A	0.6268	0.5288	0.2886	0.061*	
H8B	0.6088	0.3736	0.3202	0.061*	
C9	0.73237 (16)	0.3465 (2)	0.26119 (6)	0.0350 (4)	
H9	0.8056	0.4119	0.2695	0.042*	
C10	0.70612 (14)	0.34308 (18)	0.20101 (6)	0.0271 (3)	
C11	0.71732 (14)	0.50788 (19)	0.17824 (7)	0.0304 (3)	
H11A	0.6813	0.5084	0.1430	0.036*	
H11B	0.6696	0.5799	0.1987	0.036*	
N1	0.84734 (12)	0.56261 (15)	0.17741 (5)	0.0292 (3)	
C13	0.91738 (15)	0.46072 (19)	0.14329 (6)	0.0318 (3)	
H13A	1.0008	0.5028	0.1401	0.038*	
H13B	0.8767	0.4602	0.1089	0.038*	
C14	0.92727 (14)	0.29212 (18)	0.16352 (6)	0.0281 (3)	
O2	0.77004 (12)	0.09535 (14)	0.16831 (5)	0.0386 (3)	
C16	1.01479 (16)	0.2846 (2)	0.21325 (7)	0.0353 (4)	
H16	0.9936	0.3678	0.2375	0.042*	
C17	1.14797 (17)	0.3050 (2)	0.19615 (9)	0.0494 (5)	
H17A	1.2058	0.2529	0.2205	0.059*	
H17B	1.1694	0.4150	0.1952	0.059*	
C18	0.99011 (15)	0.1806 (2)	0.12465 (7)	0.0377 (4)	
H18	0.9768	0.0736	0.1368	0.045*	
C19	0.94465 (18)	0.1872 (2)	0.06847 (8)	0.0409 (4)	
C20	0.8536 (2)	0.0833 (3)	0.05035 (9)	0.0619 (6)	
H20	0.8218	0.0099	0.0730	0.074*	
C21	0.8096 (3)	0.0873 (4)	−0.00077 (10)	0.0759 (8)	
H21	0.7479	0.0171	−0.0119	0.091*	
C22	0.8553 (3)	0.1937 (3)	−0.03593 (9)	0.0641 (7)	
C23	0.9493 (4)	0.2889 (4)	−0.01808 (10)	0.0872 (10)	
H23	0.9846	0.3581	−0.0412	0.105*	
C24	0.9938 (3)	0.2863 (3)	0.03268 (10)	0.0762 (8)	
H24	1.0585	0.3531	0.0431	0.091*	
C25	0.8072 (4)	0.1958 (4)	−0.09210 (10)	0.0960 (11)	
H25A	0.8141	0.2997	−0.1058	0.144*	
H25B	0.7213	0.1638	−0.0940	0.144*	
H25C	0.8558	0.1254	−0.1121	0.144*	
C26	0.3467 (3)	0.3383 (6)	−0.01659 (11)	0.1097 (14)	
H26A	0.2664	0.2885	−0.0180	0.165*	
H26B	0.3999	0.2887	−0.0408	0.165*	
H26C	0.3372	0.4468	−0.0255	0.165*	
C12	0.79789 (14)	0.23038 (18)	0.17614 (6)	0.0269 (3)	
O1	0.75164 (16)	0.19543 (18)	0.28197 (6)	0.0525 (4)	
H1	0.8168	0.1593	0.2714	0.079*	
C15	0.8473 (2)	0.7232 (2)	0.15654 (8)	0.0446 (4)	
H15A	0.8097	0.7236	0.1220	0.067*	
H15B	0.9317	0.7604	0.1555	0.067*	
H15C	0.8006	0.7901	0.1785	0.067*	
O3	0.99559 (13)	0.13534 (15)	0.23626 (6)	0.0469 (4)	
H3A	1.0500	0.1195	0.2590	0.070*	
S1	0.48173 (4)	0.36957 (7)	0.24461 (2)	0.04803 (15)	
S2	1.15760 (5)	0.22095 (9)	0.13244 (3)	0.0704 (2)	

### (I) 4,11-Dihydroxy-13-methyl-1,8-di-p-tolyl-2,9-dithia-13-azadispiro[4.1.47.35]tetradecan-6-one Atomic displacement parameters (Å^2^)

**Table:**

	*U*^11^	*U*^22^	*U*^33^	*U*^12^	*U*^13^	*U*^23^
C1	0.0418 (12)	0.126 (3)	0.0471 (12)	−0.0248 (14)	−0.0074 (10)	0.0022 (15)
C2	0.0447 (12)	0.0852 (18)	0.0676 (15)	−0.0093 (12)	−0.0171 (11)	0.0202 (14)
C3	0.0368 (10)	0.0577 (13)	0.0560 (12)	−0.0038 (9)	−0.0094 (8)	0.0011 (10)
C4	0.0253 (7)	0.0494 (11)	0.0442 (10)	−0.0069 (7)	−0.0038 (7)	−0.0061 (8)
C5	0.0459 (11)	0.0785 (17)	0.0628 (14)	0.0043 (11)	−0.0140 (10)	−0.0290 (13)
C6	0.0547 (14)	0.124 (3)	0.0605 (15)	−0.0036 (16)	−0.0079 (12)	−0.0399 (17)
C7	0.0265 (7)	0.0333 (8)	0.0399 (9)	−0.0033 (6)	0.0006 (6)	−0.0029 (7)
C8	0.0446 (10)	0.0681 (14)	0.0391 (10)	0.0032 (10)	0.0008 (8)	−0.0118 (10)
C9	0.0376 (8)	0.0361 (9)	0.0309 (8)	0.0005 (7)	−0.0045 (7)	0.0004 (7)
C10	0.0253 (7)	0.0252 (7)	0.0306 (7)	−0.0007 (6)	−0.0032 (6)	0.0002 (6)
C11	0.0275 (7)	0.0262 (8)	0.0369 (8)	0.0019 (6)	−0.0057 (6)	0.0019 (6)
N1	0.0308 (6)	0.0204 (6)	0.0356 (7)	−0.0021 (5)	−0.0067 (5)	0.0029 (5)
C13	0.0320 (8)	0.0275 (8)	0.0356 (8)	−0.0019 (6)	−0.0010 (6)	0.0045 (6)
C14	0.0254 (7)	0.0243 (7)	0.0343 (8)	−0.0003 (6)	−0.0039 (6)	−0.0002 (6)
O2	0.0362 (6)	0.0242 (6)	0.0553 (8)	−0.0033 (5)	−0.0002 (5)	−0.0032 (5)
C16	0.0311 (8)	0.0267 (8)	0.0468 (10)	0.0002 (6)	−0.0131 (7)	0.0022 (7)
C17	0.0302 (9)	0.0431 (11)	0.0735 (14)	−0.0027 (8)	−0.0142 (9)	−0.0014 (10)
C18	0.0286 (8)	0.0345 (9)	0.0501 (10)	−0.0001 (7)	0.0036 (7)	−0.0061 (8)
C19	0.0434 (9)	0.0361 (9)	0.0439 (10)	−0.0021 (8)	0.0114 (8)	−0.0066 (7)
C20	0.0665 (14)	0.0687 (15)	0.0501 (12)	−0.0223 (12)	−0.0034 (10)	0.0051 (11)
C21	0.0777 (18)	0.093 (2)	0.0565 (14)	−0.0207 (16)	−0.0076 (13)	−0.0030 (14)
C22	0.0916 (19)	0.0608 (15)	0.0403 (11)	0.0116 (14)	0.0070 (11)	−0.0041 (10)
C23	0.145 (3)	0.0724 (18)	0.0464 (13)	−0.034 (2)	0.0259 (16)	−0.0004 (13)
C24	0.102 (2)	0.0764 (18)	0.0517 (13)	−0.0419 (16)	0.0238 (14)	−0.0095 (12)
C25	0.152 (3)	0.091 (2)	0.0439 (14)	0.020 (2)	−0.0038 (17)	−0.0071 (14)
C26	0.079 (2)	0.196 (4)	0.0527 (16)	−0.046 (2)	−0.0196 (14)	0.019 (2)
C12	0.0272 (7)	0.0241 (7)	0.0289 (7)	0.0000 (6)	−0.0057 (6)	0.0019 (6)
O1	0.0690 (10)	0.0477 (8)	0.0404 (7)	0.0051 (7)	−0.0010 (7)	0.0140 (6)
C15	0.0531 (11)	0.0247 (8)	0.0552 (11)	−0.0025 (8)	−0.0083 (9)	0.0095 (8)
O3	0.0449 (7)	0.0321 (7)	0.0614 (9)	−0.0024 (6)	−0.0254 (6)	0.0127 (6)
S1	0.0324 (2)	0.0625 (3)	0.0495 (3)	−0.0011 (2)	0.00694 (19)	−0.0103 (2)
S2	0.0279 (2)	0.0881 (5)	0.0956 (5)	−0.0015 (3)	0.0089 (3)	−0.0326 (4)

### (I) 4,11-Dihydroxy-13-methyl-1,8-di-p-tolyl-2,9-dithia-13-azadispiro[4.1.47.35]tetradecan-6-one Geometric parameters (Å, º)

**Table:**

C1—C2	1.372 (4)	C14—C16	1.555 (2)
C1—C6	1.382 (5)	O2—C12	1.2082 (19)
C1—C26	1.516 (4)	C16—O3	1.426 (2)
C2—C3	1.387 (3)	C16—C17	1.521 (3)
C2—H2	0.9300	C16—H16	0.9800
C3—C4	1.387 (3)	C17—S2	1.795 (2)
C3—H3	0.9300	C17—H17A	0.9700
C4—C5	1.383 (3)	C17—H17B	0.9700
C4—C7	1.513 (2)	C18—C19	1.505 (3)
C5—C6	1.384 (4)	C18—S2	1.8306 (18)
C5—H5	0.9300	C18—H18	0.9800
C6—H6	0.9300	C19—C24	1.372 (3)
C7—C10	1.556 (2)	C19—C20	1.386 (3)
C7—S1	1.8260 (18)	C20—C21	1.377 (3)
C7—H7	0.9800	C20—H20	0.9300
C8—C9	1.524 (3)	C21—C22	1.386 (4)
C8—S1	1.808 (2)	C21—H21	0.9300
C8—H8A	0.9700	C22—C23	1.360 (4)
C8—H8B	0.9700	C22—C25	1.512 (4)
C9—O1	1.411 (2)	C23—C24	1.370 (4)
C9—C10	1.560 (2)	C23—H23	0.9300
C9—H9	0.9800	C24—H24	0.9300
C10—C11	1.533 (2)	C25—H25A	0.9600
C10—C12	1.535 (2)	C25—H25B	0.9600
C11—N1	1.471 (2)	C25—H25C	0.9600
C11—H11A	0.9700	C26—H26A	0.9600
C11—H11B	0.9700	C26—H26B	0.9600
N1—C13	1.465 (2)	C26—H26C	0.9600
N1—C15	1.475 (2)	O1—H1	0.8200
C13—C14	1.536 (2)	C15—H15A	0.9600
C13—H13A	0.9700	C15—H15B	0.9600
C13—H13B	0.9700	C15—H15C	0.9600
C14—C12	1.531 (2)	O3—H3A	0.8200
C14—C18	1.555 (2)		
			
C2—C1—C6	117.0 (2)	C18—C14—C16	103.88 (13)
C2—C1—C26	121.1 (3)	O3—C16—C17	112.07 (15)
C6—C1—C26	121.8 (3)	O3—C16—C14	106.59 (13)
C1—C2—C3	121.8 (3)	C17—C16—C14	107.41 (15)
C1—C2—H2	119.1	O3—C16—H16	110.2
C3—C2—H2	119.1	C17—C16—H16	110.2
C2—C3—C4	120.8 (2)	C14—C16—H16	110.2
C2—C3—H3	119.6	C16—C17—S2	107.92 (13)
C4—C3—H3	119.6	C16—C17—H17A	110.1
C5—C4—C3	117.6 (2)	S2—C17—H17A	110.1
C5—C4—C7	118.93 (19)	C16—C17—H17B	110.1
C3—C4—C7	123.51 (18)	S2—C17—H17B	110.1
C4—C5—C6	120.7 (3)	H17A—C17—H17B	108.4
C4—C5—H5	119.6	C19—C18—C14	117.47 (15)
C6—C5—H5	119.6	C19—C18—S2	112.00 (13)
C1—C6—C5	122.0 (3)	C14—C18—S2	105.22 (11)
C1—C6—H6	119.0	C19—C18—H18	107.2
C5—C6—H6	119.0	C14—C18—H18	107.2
C4—C7—C10	116.18 (14)	S2—C18—H18	107.2
C4—C7—S1	112.50 (12)	C24—C19—C20	117.1 (2)
C10—C7—S1	105.91 (11)	C24—C19—C18	123.24 (19)
C4—C7—H7	107.3	C20—C19—C18	119.57 (18)
C10—C7—H7	107.3	C21—C20—C19	120.8 (2)
S1—C7—H7	107.3	C21—C20—H20	119.6
C9—C8—S1	108.49 (13)	C19—C20—H20	119.6
C9—C8—H8A	110.0	C20—C21—C22	121.5 (3)
S1—C8—H8A	110.0	C20—C21—H21	119.3
C9—C8—H8B	110.0	C22—C21—H21	119.3
S1—C8—H8B	110.0	C23—C22—C21	116.7 (2)
H8A—C8—H8B	108.4	C23—C22—C25	122.2 (3)
O1—C9—C8	108.14 (16)	C21—C22—C25	121.0 (3)
O1—C9—C10	112.04 (14)	C22—C23—C24	122.4 (2)
C8—C9—C10	107.47 (14)	C22—C23—H23	118.8
O1—C9—H9	109.7	C24—C23—H23	118.8
C8—C9—H9	109.7	C23—C24—C19	121.3 (3)
C10—C9—H9	109.7	C23—C24—H24	119.3
C11—C10—C12	110.93 (13)	C19—C24—H24	119.3
C11—C10—C7	111.86 (13)	C22—C25—H25A	109.5
C12—C10—C7	109.54 (13)	C22—C25—H25B	109.5
C11—C10—C9	110.30 (13)	H25A—C25—H25B	109.5
C12—C10—C9	109.40 (12)	C22—C25—H25C	109.5
C7—C10—C9	104.61 (13)	H25A—C25—H25C	109.5
N1—C11—C10	112.80 (12)	H25B—C25—H25C	109.5
N1—C11—H11A	109.0	C1—C26—H26A	109.5
C10—C11—H11A	109.0	C1—C26—H26B	109.5
N1—C11—H11B	109.0	H26A—C26—H26B	109.5
C10—C11—H11B	109.0	C1—C26—H26C	109.5
H11A—C11—H11B	107.8	H26A—C26—H26C	109.5
C13—N1—C11	109.16 (12)	H26B—C26—H26C	109.5
C13—N1—C15	109.21 (14)	O2—C12—C14	120.96 (14)
C11—N1—C15	108.43 (13)	O2—C12—C10	120.79 (14)
N1—C13—C14	112.73 (13)	C14—C12—C10	118.21 (13)
N1—C13—H13A	109.0	C9—O1—H1	109.5
C14—C13—H13A	109.0	N1—C15—H15A	109.5
N1—C13—H13B	109.0	N1—C15—H15B	109.5
C14—C13—H13B	109.0	H15A—C15—H15B	109.5
H13A—C13—H13B	107.8	N1—C15—H15C	109.5
C12—C14—C13	110.21 (12)	H15A—C15—H15C	109.5
C12—C14—C18	110.14 (13)	H15B—C15—H15C	109.5
C13—C14—C18	112.64 (14)	C16—O3—H3A	109.5
C12—C14—C16	109.42 (13)	C8—S1—C7	94.46 (8)
C13—C14—C16	110.36 (13)	C17—S2—C18	94.71 (9)
			
C6—C1—C2—C3	0.7 (4)	C13—C14—C16—C17	−73.29 (17)
C26—C1—C2—C3	178.7 (2)	C18—C14—C16—C17	47.69 (17)
C1—C2—C3—C4	0.5 (3)	O3—C16—C17—S2	84.70 (17)
C2—C3—C4—C5	−1.9 (3)	C14—C16—C17—S2	−32.04 (17)
C2—C3—C4—C7	177.32 (19)	C12—C14—C18—C19	75.91 (18)
C3—C4—C5—C6	2.2 (3)	C13—C14—C18—C19	−47.6 (2)
C7—C4—C5—C6	−177.1 (2)	C16—C14—C18—C19	−167.00 (15)
C2—C1—C6—C5	−0.4 (4)	C12—C14—C18—S2	−158.70 (11)
C26—C1—C6—C5	−178.3 (3)	C13—C14—C18—S2	77.82 (15)
C4—C5—C6—C1	−1.1 (4)	C16—C14—C18—S2	−41.61 (15)
C5—C4—C7—C10	92.4 (2)	C14—C18—C19—C24	88.9 (3)
C3—C4—C7—C10	−86.9 (2)	S2—C18—C19—C24	−33.0 (3)
C5—C4—C7—S1	−145.32 (17)	C14—C18—C19—C20	−94.8 (2)
C3—C4—C7—S1	35.4 (2)	S2—C18—C19—C20	143.26 (19)
S1—C8—C9—O1	−91.31 (17)	C24—C19—C20—C21	−3.9 (4)
S1—C8—C9—C10	29.8 (2)	C18—C19—C20—C21	179.6 (2)
C4—C7—C10—C11	46.91 (19)	C19—C20—C21—C22	0.6 (5)
S1—C7—C10—C11	−78.80 (14)	C20—C21—C22—C23	2.9 (5)
C4—C7—C10—C12	−76.51 (18)	C20—C21—C22—C25	179.4 (3)
S1—C7—C10—C12	157.79 (11)	C21—C22—C23—C24	−3.0 (5)
C4—C7—C10—C9	166.31 (15)	C25—C22—C23—C24	−179.4 (3)
S1—C7—C10—C9	40.61 (14)	C22—C23—C24—C19	−0.4 (5)
O1—C9—C10—C11	−166.28 (14)	C20—C19—C24—C23	3.9 (4)
C8—C9—C10—C11	75.07 (18)	C18—C19—C24—C23	−179.8 (3)
O1—C9—C10—C12	−44.00 (18)	C13—C14—C12—O2	144.04 (15)
C8—C9—C10—C12	−162.66 (15)	C18—C14—C12—O2	19.1 (2)
O1—C9—C10—C7	73.28 (17)	C16—C14—C12—O2	−94.44 (18)
C8—C9—C10—C7	−45.38 (18)	C13—C14—C12—C10	−38.32 (18)
C12—C10—C11—N1	−48.75 (17)	C18—C14—C12—C10	−163.20 (13)
C7—C10—C11—N1	−171.38 (13)	C16—C14—C12—C10	83.21 (16)
C9—C10—C11—N1	72.63 (17)	C11—C10—C12—O2	−144.83 (15)
C10—C11—N1—C13	62.95 (16)	C7—C10—C12—O2	−20.9 (2)
C10—C11—N1—C15	−178.17 (14)	C9—C10—C12—O2	93.27 (17)
C11—N1—C13—C14	−64.23 (16)	C11—C10—C12—C14	37.52 (18)
C15—N1—C13—C14	177.37 (13)	C7—C10—C12—C14	161.49 (13)
N1—C13—C14—C12	50.91 (17)	C9—C10—C12—C14	−84.38 (16)
N1—C13—C14—C18	174.35 (13)	C9—C8—S1—C7	−4.88 (16)
N1—C13—C14—C16	−70.05 (17)	C4—C7—S1—C8	−149.17 (14)
C12—C14—C16—O3	44.99 (18)	C10—C7—S1—C8	−21.24 (14)
C13—C14—C16—O3	166.42 (14)	C16—C17—S2—C18	6.01 (15)
C18—C14—C16—O3	−72.60 (17)	C19—C18—S2—C17	150.01 (14)
C12—C14—C16—C17	165.27 (14)	C14—C18—S2—C17	21.28 (14)

### (I) 4,11-Dihydroxy-13-methyl-1,8-di-p-tolyl-2,9-dithia-13-azadispiro[4.1.47.35]tetradecan-6-one Hydrogen-bond geometry (Å, º)

**Table:**

*D*—H···*A*	*D*—H	H···*A*	*D*···*A*	*D*—H···*A*
O1—H1···O3	0.82	2.16	2.955 (2)	163
O3—H3*A*···N1^i^	0.82	1.99	2.798 (2)	167
C16—H16···O3^ii^	0.98	2.39	3.273 (2)	150

Symmetry codes: (i) −*x*+2, *y*−1/2, −*z*+1/2; (ii) −*x*+2, *y*+1/2, −*z*+1/2.

### (II) 13-Benzyl-4,11-dihydroxy-1,8-bis(4-methylphenyl)-2,9-dithia-13-azadispiro[4.1.47.35]tetradecan-6-one Crystal data

**Table:**

C_32_H_35_NO_3_S_2_	*Z* = 2
*M**_r_* = 545.73	*F*(000) = 580
Triclinic, *P*1	*D*_x_ = 1.301 Mg m^−^^3^
Hall symbol: -P 1	Mo *K*α radiation, λ = 0.71073 Å
*a* = 9.9803 (6) Å	Cell parameters from 5668 reflections
*b* = 11.7773 (8) Å	θ = 1.7–26.4°
*c* = 13.6506 (14) Å	µ = 0.23 mm^−^^1^
α = 105.524 (5)°	*T* = 293 K
β = 107.215 (5)°	Block, colourless
γ = 103.087 (4)°	0.20 × 0.15 × 0.10 mm
*V* = 1392.90 (19) Å^3^	

### (II) 13-Benzyl-4,11-dihydroxy-1,8-bis(4-methylphenyl)-2,9-dithia-13-azadispiro[4.1.47.35]tetradecan-6-one Data collection

**Table:**

Bruker SMART APEXII area-detector diffractometer	5668 independent reflections
Radiation source: fine-focus sealed tube	4271 reflections with *I* > 2σ(*I*)
Graphite monochromator	*R*_int_ = 0.035
ω and φ scans	θ_max_ = 26.4°, θ_min_ = 1.7°
Absorption correction: multi-scan (*SADABS*; Bruker, 2008)	*h* = −12→12
*T*_min_ = 0.956, *T*_max_ = 0.978	*k* = −14→14
20239 measured reflections	*l* = −16→16

### (II) 13-Benzyl-4,11-dihydroxy-1,8-bis(4-methylphenyl)-2,9-dithia-13-azadispiro[4.1.47.35]tetradecan-6-one Refinement

**Table:**

Refinement on *F*^2^	Primary atom site location: structure-invariant direct methods
Least-squares matrix: full	Secondary atom site location: difference Fourier map
*R*[*F*^2^ > 2σ(*F*^2^)] = 0.045	Hydrogen site location: inferred from neighbouring sites
*wR*(*F*^2^) = 0.136	H atoms treated by a mixture of independent and constrained refinement
*S* = 1.07	*w* = 1/[σ^2^(*F*_o_^2^) + (0.0561*P*)^2^ + 0.7912*P*] where *P* = (*F*_o_^2^ + 2*F*_c_^2^)/3
5668 reflections	(Δ/σ)_max_ = 0.039
349 parameters	Δρ_max_ = 0.30 e Å^−^^3^
1 restraint	Δρ_min_ = −0.50 e Å^−^^3^

### (II) 13-Benzyl-4,11-dihydroxy-1,8-bis(4-methylphenyl)-2,9-dithia-13-azadispiro[4.1.47.35]tetradecan-6-one Special details

**Table:**

Geometry. All esds (except the esd in the dihedral angle between two l.s. planes) are estimated using the full covariance matrix. The cell esds are taken into account individually in the estimation of esds in distances, angles and torsion angles; correlations between esds in cell parameters are only used when they are defined by crystal symmetry. An approximate (isotropic) treatment of cell esds is used for estimating esds involving l.s. planes.
Refinement. Refinement of F^2^ against ALL reflections. The weighted R-factor wR and goodness of fit S are based on F^2^, conventional R-factors R are based on F, with F set to zero for negative F^2^. The threshold expression of F^2^ > 2sigma(F^2^) is used only for calculating R-factors(gt) etc. and is not relevant to the choice of reflections for refinement. R-factors based on F^2^ are statistically about twice as large as those based on F, and R- factors based on ALL data will be even larger.

### (II) 13-Benzyl-4,11-dihydroxy-1,8-bis(4-methylphenyl)-2,9-dithia-13-azadispiro[4.1.47.35]tetradecan-6-one Fractional atomic coordinates and isotropic or equivalent isotropic displacement parameters (Å^2^)

**Table:**

	*x*	*y*	*z*	*U*_iso_*/*U*_eq_	
C1	0.8797 (3)	0.6378 (2)	0.1804 (2)	0.0523 (6)	
C2	0.7697 (3)	0.5580 (2)	0.1950 (2)	0.0553 (6)	
H2	0.7056	0.5894	0.2227	0.066*	
C3	0.7519 (2)	0.4328 (2)	0.1697 (2)	0.0476 (5)	
H3	0.6769	0.3818	0.1814	0.057*	
C4	0.8438 (2)	0.38122 (19)	0.12686 (17)	0.0356 (4)	
C5	0.9562 (2)	0.4619 (2)	0.1134 (2)	0.0447 (5)	
H5	1.0209	0.4309	0.0861	0.054*	
C6	0.9737 (3)	0.5873 (2)	0.1399 (2)	0.0528 (6)	
H6	1.0503	0.6391	0.1304	0.063*	
C7	0.8278 (2)	0.24410 (19)	0.09546 (17)	0.0373 (4)	
H7	0.8458	0.2197	0.0273	0.045*	
C8	0.7035 (2)	0.0592 (2)	0.1530 (2)	0.0504 (6)	
H8A	0.6998	0.0943	0.2246	0.061*	
H8B	0.6416	−0.0281	0.1200	0.061*	
C9	0.8627 (2)	0.07220 (19)	0.16339 (18)	0.0406 (5)	
H9	0.9133	0.0549	0.2283	0.049*	
C10	0.9391 (2)	0.21064 (17)	0.18060 (16)	0.0319 (4)	
C11	0.9745 (2)	0.29249 (19)	0.29918 (16)	0.0353 (4)	
H11A	1.0086	0.3800	0.3082	0.042*	
H11B	0.8854	0.2764	0.3156	0.042*	
C12	1.2306 (2)	0.31299 (19)	0.36365 (17)	0.0367 (4)	
H12A	1.3110	0.3084	0.4217	0.044*	
H12B	1.2495	0.3999	0.3709	0.044*	
C13	1.2257 (2)	0.23660 (18)	0.25115 (16)	0.0332 (4)	
C14	1.0832 (2)	0.22128 (17)	0.15850 (16)	0.0326 (4)	
C15	1.2342 (3)	0.1062 (2)	0.25259 (19)	0.0434 (5)	
H15	1.1806	0.0807	0.2969	0.052*	
C16	1.3981 (3)	0.1214 (3)	0.3083 (3)	0.0771 (9)	
H16A	1.4221	0.0556	0.2641	0.093*	
H16B	1.4155	0.1138	0.3797	0.093*	
C17	1.3638 (2)	0.2929 (2)	0.22724 (18)	0.0370 (4)	
H17	1.3493	0.2396	0.1536	0.044*	
C18	1.3963 (2)	0.4256 (2)	0.22939 (17)	0.0359 (4)	
C19	1.3530 (3)	0.4447 (2)	0.1302 (2)	0.0473 (5)	
H19	1.3093	0.3764	0.0645	0.057*	
C20	1.3742 (3)	0.5639 (2)	0.1281 (2)	0.0549 (6)	
H20	1.3431	0.5741	0.0607	0.066*	
C21	1.4403 (3)	0.6678 (2)	0.2232 (2)	0.0517 (6)	
C22	1.4881 (2)	0.6492 (2)	0.3219 (2)	0.0462 (5)	
H22	1.5353	0.7179	0.3872	0.055*	
C23	1.4670 (2)	0.5305 (2)	0.32530 (18)	0.0407 (5)	
H23	1.5007	0.5207	0.3927	0.049*	
C24	1.0994 (2)	0.3146 (2)	0.48788 (17)	0.0419 (5)	
H24A	1.1076	0.4020	0.5070	0.050*	
H24B	1.1883	0.3091	0.5373	0.050*	
C25	0.9655 (2)	0.2443 (2)	0.50273 (17)	0.0414 (5)	
C26	0.8899 (3)	0.3059 (3)	0.5544 (2)	0.0653 (7)	
H26	0.9200	0.3926	0.5789	0.078*	
C27	0.7694 (4)	0.2405 (5)	0.5704 (3)	0.0973 (13)	
H27	0.7191	0.2835	0.6055	0.117*	
C28	0.7242 (4)	0.1134 (5)	0.5352 (3)	0.1005 (14)	
H28	0.6438	0.0698	0.5468	0.121*	
C29	0.7973 (4)	0.0500 (4)	0.4825 (3)	0.0861 (11)	
H29	0.7659	−0.0368	0.4575	0.103*	
C30	0.9178 (3)	0.1153 (3)	0.4668 (2)	0.0587 (6)	
H30	0.9676	0.0719	0.4314	0.070*	
C31	0.8977 (4)	0.7745 (3)	0.2073 (3)	0.0809 (10)	
H31A	0.9992	0.8238	0.2536	0.121*	
H31B	0.8339	0.7951	0.2448	0.121*	
H31C	0.8714	0.7918	0.1405	0.121*	
C32	1.4589 (4)	0.7978 (3)	0.2204 (3)	0.0879 (11)	
H32A	1.3655	0.8123	0.2075	0.132*	
H32B	1.4918	0.8054	0.1623	0.132*	
H32C	1.5313	0.8582	0.2894	0.132*	
N1	1.08991 (17)	0.26497 (15)	0.37491 (13)	0.0336 (4)	
O1	0.8638 (2)	−0.01340 (14)	0.06942 (15)	0.0594 (5)	
H1	0.9499	−0.0057	0.0757	0.089*	
O2	1.08398 (17)	0.21434 (14)	0.06883 (12)	0.0427 (4)	
O3	1.1736 (2)	0.01162 (16)	0.14756 (17)	0.0606 (5)	
S1	0.64069 (6)	0.14300 (6)	0.06705 (6)	0.0616 (2)	
S2	1.51660 (6)	0.27141 (6)	0.32504 (6)	0.05307 (19)	
H3A	1.170 (10)	0.013 (8)	0.087 (3)	0.28 (4)*	

### (II) 13-Benzyl-4,11-dihydroxy-1,8-bis(4-methylphenyl)-2,9-dithia-13-azadispiro[4.1.47.35]tetradecan-6-one Atomic displacement parameters (Å^2^)

**Table:**

	*U*^11^	*U*^22^	*U*^33^	*U*^12^	*U*^13^	*U*^23^
C1	0.0624 (15)	0.0476 (13)	0.0453 (14)	0.0226 (12)	0.0122 (12)	0.0202 (11)
C2	0.0549 (15)	0.0619 (15)	0.0620 (16)	0.0323 (13)	0.0272 (13)	0.0255 (13)
C3	0.0361 (11)	0.0606 (14)	0.0571 (15)	0.0189 (10)	0.0224 (11)	0.0301 (12)
C4	0.0296 (10)	0.0438 (11)	0.0346 (11)	0.0125 (8)	0.0090 (8)	0.0193 (9)
C5	0.0390 (12)	0.0499 (12)	0.0545 (14)	0.0153 (10)	0.0240 (11)	0.0255 (11)
C6	0.0541 (14)	0.0488 (13)	0.0579 (15)	0.0095 (11)	0.0229 (12)	0.0272 (12)
C7	0.0288 (10)	0.0433 (11)	0.0362 (11)	0.0075 (8)	0.0079 (8)	0.0174 (9)
C8	0.0362 (12)	0.0482 (13)	0.0552 (15)	−0.0008 (10)	0.0116 (11)	0.0197 (11)
C9	0.0395 (11)	0.0353 (10)	0.0405 (12)	0.0049 (9)	0.0112 (9)	0.0144 (9)
C10	0.0269 (9)	0.0329 (10)	0.0334 (10)	0.0073 (8)	0.0091 (8)	0.0130 (8)
C11	0.0304 (10)	0.0392 (10)	0.0348 (11)	0.0110 (8)	0.0110 (8)	0.0130 (9)
C12	0.0284 (10)	0.0417 (11)	0.0358 (11)	0.0070 (8)	0.0100 (9)	0.0137 (9)
C13	0.0297 (10)	0.0362 (10)	0.0371 (11)	0.0121 (8)	0.0132 (8)	0.0169 (9)
C14	0.0329 (10)	0.0281 (9)	0.0338 (11)	0.0074 (8)	0.0112 (8)	0.0105 (8)
C15	0.0480 (13)	0.0407 (11)	0.0486 (13)	0.0180 (10)	0.0201 (11)	0.0223 (10)
C16	0.0608 (17)	0.0567 (16)	0.111 (3)	0.0266 (14)	0.0117 (17)	0.0439 (17)
C17	0.0308 (10)	0.0460 (11)	0.0412 (12)	0.0162 (9)	0.0170 (9)	0.0197 (9)
C18	0.0243 (9)	0.0473 (11)	0.0417 (12)	0.0123 (8)	0.0154 (9)	0.0211 (10)
C19	0.0397 (12)	0.0549 (13)	0.0420 (13)	0.0062 (10)	0.0117 (10)	0.0216 (11)
C20	0.0470 (13)	0.0650 (16)	0.0535 (15)	0.0112 (12)	0.0117 (12)	0.0370 (13)
C21	0.0387 (12)	0.0509 (13)	0.0675 (17)	0.0115 (10)	0.0173 (12)	0.0303 (13)
C22	0.0345 (11)	0.0456 (12)	0.0534 (14)	0.0078 (9)	0.0158 (10)	0.0159 (11)
C23	0.0293 (10)	0.0534 (13)	0.0411 (12)	0.0103 (9)	0.0137 (9)	0.0220 (10)
C24	0.0373 (11)	0.0495 (12)	0.0340 (11)	0.0089 (9)	0.0123 (9)	0.0139 (9)
C25	0.0357 (11)	0.0583 (13)	0.0329 (11)	0.0170 (10)	0.0127 (9)	0.0196 (10)
C26	0.0576 (16)	0.093 (2)	0.0606 (17)	0.0383 (15)	0.0315 (14)	0.0303 (15)
C27	0.062 (2)	0.177 (4)	0.094 (3)	0.060 (3)	0.054 (2)	0.068 (3)
C28	0.0418 (16)	0.175 (4)	0.093 (3)	0.012 (2)	0.0281 (18)	0.077 (3)
C29	0.069 (2)	0.092 (2)	0.083 (2)	−0.0077 (18)	0.0185 (18)	0.0480 (19)
C30	0.0553 (15)	0.0636 (16)	0.0601 (16)	0.0120 (12)	0.0257 (13)	0.0288 (13)
C31	0.110 (3)	0.0518 (16)	0.078 (2)	0.0338 (17)	0.025 (2)	0.0267 (15)
C32	0.090 (2)	0.0593 (18)	0.110 (3)	0.0150 (17)	0.023 (2)	0.0478 (19)
N1	0.0278 (8)	0.0414 (9)	0.0312 (9)	0.0091 (7)	0.0107 (7)	0.0150 (7)
O1	0.0632 (11)	0.0353 (8)	0.0706 (12)	0.0061 (8)	0.0314 (10)	0.0070 (8)
O2	0.0425 (8)	0.0520 (9)	0.0334 (8)	0.0138 (7)	0.0155 (7)	0.0153 (7)
O3	0.0809 (13)	0.0417 (9)	0.0590 (12)	0.0265 (9)	0.0248 (10)	0.0146 (8)
S1	0.0285 (3)	0.0585 (4)	0.0774 (5)	−0.0016 (3)	−0.0024 (3)	0.0320 (4)
S2	0.0349 (3)	0.0693 (4)	0.0699 (4)	0.0264 (3)	0.0209 (3)	0.0390 (3)

### (II) 13-Benzyl-4,11-dihydroxy-1,8-bis(4-methylphenyl)-2,9-dithia-13-azadispiro[4.1.47.35]tetradecan-6-one Geometric parameters (Å, º)

**Table:**

C1—C2	1.375 (4)	C16—H16A	0.9700
C1—C6	1.387 (4)	C16—H16B	0.9700
C1—C31	1.508 (4)	C17—C18	1.513 (3)
C2—C3	1.377 (3)	C17—S2	1.823 (2)
C2—H2	0.9300	C17—H17	0.9800
C3—C4	1.390 (3)	C18—C19	1.389 (3)
C3—H3	0.9300	C18—C23	1.390 (3)
C4—C5	1.389 (3)	C19—C20	1.380 (3)
C4—C7	1.513 (3)	C19—H19	0.9300
C5—C6	1.378 (3)	C20—C21	1.377 (4)
C5—H5	0.9300	C20—H20	0.9300
C6—H6	0.9300	C21—C22	1.385 (3)
C7—C10	1.555 (3)	C21—C32	1.512 (4)
C7—S1	1.837 (2)	C22—C23	1.381 (3)
C7—H7	0.9800	C22—H22	0.9300
C8—C9	1.520 (3)	C23—H23	0.9300
C8—S1	1.791 (3)	C24—N1	1.461 (3)
C8—H8A	0.9700	C24—C25	1.504 (3)
C8—H8B	0.9700	C24—H24A	0.9700
C9—O1	1.408 (3)	C24—H24B	0.9700
C9—C10	1.556 (3)	C25—C26	1.371 (3)
C9—H9	0.9800	C25—C30	1.382 (3)
C10—C11	1.532 (3)	C26—C27	1.384 (5)
C10—C14	1.539 (3)	C26—H26	0.9300
C11—N1	1.464 (3)	C27—C28	1.362 (6)
C11—H11A	0.9700	C27—H27	0.9300
C11—H11B	0.9700	C28—C29	1.370 (6)
C12—N1	1.460 (2)	C28—H28	0.9300
C12—C13	1.538 (3)	C29—C30	1.382 (4)
C12—H12A	0.9700	C29—H29	0.9300
C12—H12B	0.9700	C30—H30	0.9300
C13—C14	1.534 (3)	C31—H31A	0.9600
C13—C17	1.553 (3)	C31—H31B	0.9600
C13—C15	1.562 (3)	C31—H31C	0.9600
C14—O2	1.207 (2)	C32—H32A	0.9600
C15—O3	1.410 (3)	C32—H32B	0.9600
C15—C16	1.531 (4)	C32—H32C	0.9600
C15—H15	0.9800	O1—H1	0.8200
C16—S2	1.803 (3)	O3—H3A	0.82 (2)
			
C2—C1—C6	117.2 (2)	S2—C16—H16A	109.5
C2—C1—C31	121.5 (3)	C15—C16—H16B	109.5
C6—C1—C31	121.3 (3)	S2—C16—H16B	109.5
C1—C2—C3	121.8 (2)	H16A—C16—H16B	108.1
C1—C2—H2	119.1	C18—C17—C13	117.59 (16)
C3—C2—H2	119.1	C18—C17—S2	113.06 (14)
C2—C3—C4	121.2 (2)	C13—C17—S2	104.14 (13)
C2—C3—H3	119.4	C18—C17—H17	107.2
C4—C3—H3	119.4	C13—C17—H17	107.2
C5—C4—C3	117.1 (2)	S2—C17—H17	107.2
C5—C4—C7	119.27 (19)	C19—C18—C23	117.6 (2)
C3—C4—C7	123.64 (19)	C19—C18—C17	118.6 (2)
C6—C5—C4	121.1 (2)	C23—C18—C17	123.76 (19)
C6—C5—H5	119.4	C20—C19—C18	120.8 (2)
C4—C5—H5	119.4	C20—C19—H19	119.6
C5—C6—C1	121.5 (2)	C18—C19—H19	119.6
C5—C6—H6	119.2	C21—C20—C19	121.7 (2)
C1—C6—H6	119.2	C21—C20—H20	119.2
C4—C7—C10	115.31 (16)	C19—C20—H20	119.2
C4—C7—S1	113.35 (14)	C20—C21—C22	117.6 (2)
C10—C7—S1	106.77 (13)	C20—C21—C32	121.4 (3)
C4—C7—H7	107.0	C22—C21—C32	121.0 (3)
C10—C7—H7	107.0	C23—C22—C21	121.3 (2)
S1—C7—H7	107.0	C23—C22—H22	119.3
C9—C8—S1	106.11 (15)	C21—C22—H22	119.3
C9—C8—H8A	110.5	C22—C23—C18	120.9 (2)
S1—C8—H8A	110.5	C22—C23—H23	119.5
C9—C8—H8B	110.5	C18—C23—H23	119.5
S1—C8—H8B	110.5	N1—C24—C25	111.66 (17)
H8A—C8—H8B	108.7	N1—C24—H24A	109.3
O1—C9—C8	110.11 (19)	C25—C24—H24A	109.3
O1—C9—C10	112.94 (17)	N1—C24—H24B	109.3
C8—C9—C10	106.43 (17)	C25—C24—H24B	109.3
O1—C9—H9	109.1	H24A—C24—H24B	107.9
C8—C9—H9	109.1	C26—C25—C30	118.3 (2)
C10—C9—H9	109.1	C26—C25—C24	121.2 (2)
C11—C10—C14	110.28 (15)	C30—C25—C24	120.4 (2)
C11—C10—C7	112.19 (16)	C25—C26—C27	120.8 (3)
C14—C10—C7	110.24 (16)	C25—C26—H26	119.6
C11—C10—C9	108.78 (16)	C27—C26—H26	119.6
C14—C10—C9	108.77 (16)	C28—C27—C26	120.3 (3)
C7—C10—C9	106.46 (15)	C28—C27—H27	119.9
N1—C11—C10	109.70 (16)	C26—C27—H27	119.9
N1—C11—H11A	109.7	C27—C28—C29	119.9 (3)
C10—C11—H11A	109.7	C27—C28—H28	120.1
N1—C11—H11B	109.7	C29—C28—H28	120.1
C10—C11—H11B	109.7	C28—C29—C30	119.8 (4)
H11A—C11—H11B	108.2	C28—C29—H29	120.1
N1—C12—C13	110.40 (16)	C30—C29—H29	120.1
N1—C12—H12A	109.6	C25—C30—C29	120.9 (3)
C13—C12—H12A	109.6	C25—C30—H30	119.6
N1—C12—H12B	109.6	C29—C30—H30	119.6
C13—C12—H12B	109.6	C1—C31—H31A	109.5
H12A—C12—H12B	108.1	C1—C31—H31B	109.5
C14—C13—C12	110.81 (16)	H31A—C31—H31B	109.5
C14—C13—C17	109.88 (16)	C1—C31—H31C	109.5
C12—C13—C17	113.19 (16)	H31A—C31—H31C	109.5
C14—C13—C15	110.49 (16)	H31B—C31—H31C	109.5
C12—C13—C15	108.44 (16)	C21—C32—H32A	109.5
C17—C13—C15	103.81 (16)	C21—C32—H32B	109.5
O2—C14—C13	120.01 (18)	H32A—C32—H32B	109.5
O2—C14—C10	120.85 (18)	C21—C32—H32C	109.5
C13—C14—C10	119.11 (16)	H32A—C32—H32C	109.5
O3—C15—C16	109.4 (2)	H32B—C32—H32C	109.5
O3—C15—C13	114.05 (18)	C12—N1—C24	112.54 (16)
C16—C15—C13	107.70 (18)	C12—N1—C11	108.66 (15)
O3—C15—H15	108.5	C24—N1—C11	111.57 (16)
C16—C15—H15	108.5	C9—O1—H1	109.5
C13—C15—H15	108.5	C15—O3—H3A	132 (6)
C15—C16—S2	110.68 (17)	C8—S1—C7	95.12 (10)
C15—C16—H16A	109.5	C16—S2—C17	91.17 (11)
			
C6—C1—C2—C3	−0.6 (4)	C17—C13—C15—O3	−83.2 (2)
C31—C1—C2—C3	179.4 (3)	C14—C13—C15—C16	156.2 (2)
C1—C2—C3—C4	−0.7 (4)	C12—C13—C15—C16	−82.2 (2)
C2—C3—C4—C5	1.5 (3)	C17—C13—C15—C16	38.4 (3)
C2—C3—C4—C7	−178.8 (2)	O3—C15—C16—S2	113.2 (2)
C3—C4—C5—C6	−1.0 (3)	C13—C15—C16—S2	−11.3 (3)
C7—C4—C5—C6	179.3 (2)	C14—C13—C17—C18	66.7 (2)
C4—C5—C6—C1	−0.3 (4)	C12—C13—C17—C18	−57.8 (2)
C2—C1—C6—C5	1.2 (4)	C15—C13—C17—C18	−175.15 (18)
C31—C1—C6—C5	−178.9 (2)	C14—C13—C17—S2	−167.34 (13)
C5—C4—C7—C10	78.8 (2)	C12—C13—C17—S2	68.19 (18)
C3—C4—C7—C10	−100.8 (2)	C15—C13—C17—S2	−49.17 (18)
C5—C4—C7—S1	−157.71 (17)	C13—C17—C18—C19	−99.7 (2)
C3—C4—C7—S1	22.6 (3)	S2—C17—C18—C19	138.84 (17)
S1—C8—C9—O1	−80.24 (19)	C13—C17—C18—C23	80.1 (2)
S1—C8—C9—C10	42.5 (2)	S2—C17—C18—C23	−41.3 (2)
C4—C7—C10—C11	35.2 (2)	C23—C18—C19—C20	−2.7 (3)
S1—C7—C10—C11	−91.72 (17)	C17—C18—C19—C20	177.1 (2)
C4—C7—C10—C14	−88.1 (2)	C18—C19—C20—C21	0.9 (4)
S1—C7—C10—C14	144.97 (14)	C19—C20—C21—C22	1.4 (4)
C4—C7—C10—C9	154.07 (17)	C19—C20—C21—C32	−178.1 (3)
S1—C7—C10—C9	27.17 (19)	C20—C21—C22—C23	−1.6 (3)
O1—C9—C10—C11	−163.04 (17)	C32—C21—C22—C23	177.9 (2)
C8—C9—C10—C11	76.0 (2)	C21—C22—C23—C18	−0.3 (3)
O1—C9—C10—C14	−42.9 (2)	C19—C18—C23—C22	2.5 (3)
C8—C9—C10—C14	−163.83 (17)	C17—C18—C23—C22	−177.36 (18)
O1—C9—C10—C7	75.9 (2)	N1—C24—C25—C26	−131.5 (2)
C8—C9—C10—C7	−45.1 (2)	N1—C24—C25—C30	50.1 (3)
C14—C10—C11—N1	−51.4 (2)	C30—C25—C26—C27	0.3 (4)
C7—C10—C11—N1	−174.70 (15)	C24—C25—C26—C27	−178.1 (3)
C9—C10—C11—N1	67.8 (2)	C25—C26—C27—C28	0.1 (5)
N1—C12—C13—C14	48.6 (2)	C26—C27—C28—C29	−0.7 (6)
N1—C12—C13—C17	172.50 (16)	C27—C28—C29—C30	0.8 (5)
N1—C12—C13—C15	−72.9 (2)	C26—C25—C30—C29	−0.1 (4)
C12—C13—C14—O2	148.46 (18)	C24—C25—C30—C29	178.3 (2)
C17—C13—C14—O2	22.6 (2)	C28—C29—C30—C25	−0.4 (5)
C15—C13—C14—O2	−91.3 (2)	C13—C12—N1—C24	166.97 (17)
C12—C13—C14—C10	−33.5 (2)	C13—C12—N1—C11	−69.0 (2)
C17—C13—C14—C10	−159.36 (16)	C25—C24—N1—C12	−167.72 (17)
C15—C13—C14—C10	86.7 (2)	C25—C24—N1—C11	69.8 (2)
C11—C10—C14—O2	−146.97 (18)	C10—C11—N1—C12	70.60 (19)
C7—C10—C14—O2	−22.5 (2)	C10—C11—N1—C24	−164.76 (16)
C9—C10—C14—O2	93.8 (2)	C9—C8—S1—C7	−23.05 (18)
C11—C10—C14—C13	35.0 (2)	C4—C7—S1—C8	−130.77 (16)
C7—C10—C14—C13	159.47 (16)	C10—C7—S1—C8	−2.71 (16)
C9—C10—C14—C13	−84.2 (2)	C15—C16—S2—C17	−15.5 (2)
C14—C13—C15—O3	34.5 (2)	C18—C17—S2—C16	166.52 (18)
C12—C13—C15—O3	156.18 (18)	C13—C17—S2—C16	37.74 (18)

### (II) 13-Benzyl-4,11-dihydroxy-1,8-bis(4-methylphenyl)-2,9-dithia-13-azadispiro[4.1.47.35]tetradecan-6-one Hydrogen-bond geometry (Å, º)

Cg4 and Cg5 are the centroids of the *B* (C1–C6) and *C* (C18–C23) toluyl rings, respectively.

**Table:**

*D*—H···*A*	*D*—H	H···*A*	*D*···*A*	*D*—H···*A*
O1—H1···O3	0.82	2.09	2.873 (3)	159
O3—H3*A*···O1^i^	0.82 (6)	2.06 (5)	2.880 (3)	174 (1)
C2—H2···*Cg*5^ii^	0.93	2.80	3.620 (3)	148
C20—H20···*Cg*4^iii^	0.93	2.79	3.616 (3)	149

Symmetry codes: (i) −*x*+2, −*y*, −*z*; (ii) *x*−1, *y*, *z*; (iii) −*x*+2, −*y*+1, −*z*.
